# Machine Learning Algorithm to Explore Patients With Heterogeneous Treatment Effects of Clinically Significant CMV Infection and Non‐Relapse Mortality After HSCT

**DOI:** 10.1002/jha2.70117

**Published:** 2025-08-09

**Authors:** Takashi Toya, Yujiro Nakajima, Konan Hara, Satoshi Kaito, Tetsuya Nishida, Naoyuki Uchida, Naoki Shingai, Wataru Takeda, Yukiyasu Ozawa, Masatsugu Tanaka, Satoshi Yoshihara, Yuta Katayama, Tetsuya Eto, Masashi Sawa, Shuichi Ota, Hiroyuki Ohigashi, Satoru Takada, Keisuke Kataoka, Junya Kanda, Takahiro Fukuda, Masao Ogata, Ayumi Taguchi, Yoshiko Atsuta

**Affiliations:** ^1^ Hematology Division Tokyo Metropolitan Komagome Hospital Tokyo Japan; ^2^ Department of Radiological Sciences Komazawa University Graduate School Setagaya Japan; ^3^ Department of Radiation Oncology Tokyo Metropolitan Komagome Hospital Tokyo Japan; ^4^ Department of Economics University of Arizona Tucson Arizona USA; ^5^ Department of Hematology Japanese Red Cross Aichi Medical Center Nagoya Daiichi Hospital Nagoya Japan; ^6^ Department of Hematology Federation of National Public Service Personnel Mutual Aid Associations Toranomon Hospital Tokyo Japan; ^7^ Department of Hematopoietic Stem Cell Transplantation National Cancer Center Hospital Tokyo Japan; ^8^ Department of Hematology Kanagawa Cancer Center Yokohama Japan; ^9^ Department of Respiratory Medicine and Hematology Hyogo Medical University Nishinomiya Japan; ^10^ Department of Hematology Hiroshima Red Cross Hospital & Atomic‐Bomb Survivors Hospital Hiroshima Japan; ^11^ Department of Hematology Hamanomachi Hospital Fukuoka Japan; ^12^ Department of Hematology and Oncology Anjo Kosei Hospital Anjo Japan; ^13^ Department of Hematology Sapporo Hokuyu Hospital Sapporo Japan; ^14^ Department of Hematology Hokkaido University Hospital Sapporo Japan; ^15^ Leukemia Research Center Saiseikai Maebashi Hospital Maebashi Japan; ^16^ Division of Molecular Oncology National Cancer Center Research Institute Tokyo Japan; ^17^ Division of Hematology Department of Medicine Keio University School of Medicine Shinjuku Japan; ^18^ Department of Hematology and Oncology Graduate School of Medicine Kyoto University Kyoto Japan; ^19^ Department of Medical Oncology and Hematology Oita University Faculty of Medicine Oita Japan; ^20^ Department of Gynecology The University of Tokyo Bunkyo Japan; ^21^ Gynecology Division Tokyo Metropolitan Komagome Hospital Tokyo Japan; ^22^ Japanese Data Center for Hematopoietic Cell Transplantation Nagoya Japan; ^23^ Department of Registry Science for Transplant and Cellular Therapy Aichi Medical University School of Medicine Nagakute Japan

**Keywords:** cytomegalovirus, risk factors, stem cell transplantation

## Abstract

**Introduction:**

Clinically significant cytomegalovirus infection (csCMVi) and non‐relapse mortality (NRM) remain serious concerns after allogeneic hematopoietic stem cell transplantation (HSCT), but subpopulations with heterogeneous treatment effects (HTEs) is unclear. Although machine learning (ML) algorithms have recently been applied to HSCT, the methodology has not been well elucidated.

**Methods:**

We developed a ML algorithm which combined weighting procedures and left‐truncated and right‐censored trees based on classification and regression tree algorithms to fit survival data with time‐varying covariates and competing risks comprehensively. The Japanese large‐scale registry data were applied to the algorithm to explore subpopulations with HTEs of csCMVi and NRM after HSCT. Its performance was evaluated by comparing their c‐indices with those of the conventional Fine‐Gray model.

**Results:**

A total of 10,480 patients were divided into training (75%) and test (25%) cohorts; the training cohort was used to develop the ML model. Using the model, patient CMV‐seropositivity, patient age, and acute graft‐versus‐host disease were identified as important predictors of csCMVi. In addition, the patients were successfully classified by the estimated cumulative incidence of csCMVi, which varied from 22.7% at 0.5 year to 82.7%. This model also depicts interpretable survival trees in various settings. Similarly, the patients can be also classified based on the estimated 3‐year NRM, which varied from 8.0% to 48.5%. C‐indices of the ML and the Fine‐Gray model using the test cohort showed comparable performance.

**Conclusion:**

A reliable, explainable, and interpretable ML model was developed to explore subpopulations with HTEs of csCMVi and NRM after HSCT.

**Trial Registration**: The authors have confirmed clinical trial registration is not needed for this submission

## 1 Introduction

Allogeneic hematopoietic stem cell transplantation (HSCT) is a curative treatment for many hematological diseases. However, HSCT recipients are at high risk of infectious complications, including clinically significant cytomegalovirus infection (csCMVi) [1, 2, 3, 4], and csCMVi can, directly and indirectly, increase non‐relapse mortality (NRM) [1, 2, 3, 5] and relapse risk [6, 7, 8]. Recently, universal prophylaxis with letermovir has been widely used for cytomegalovirus (CMV) seropositive HSCT recipients [9]. However, considering that approximately half of the HSCT recipients without any CMV prophylaxis eventually do not develop csCMVi [1, 2, 3, 4], elucidation of prophylaxis for csCMVi and more reliable predicting methods for CMV infection is warranted. In addition, the prediction of NRM is important for optimal patient selection. Some risk classifications have been proposed to classify the risk of CMV infection [10, 11, 12] and NRM [13, 14, 15] such as hematopoietic cell transplantation‐specific comorbidity index; however, their accuracies are suboptimal and can only sometimes be applied to individual patients.

Machine learning (ML) algorithms have recently been applied to hematology and HSCT [16, 17, 18, 19] and those studies have successfully predicted variable events such as graft‐versus‐host disease (GVHD) [20], NRM [21], and GVHD‐free, relapse‐free survival [22]. In addition, the explainability of ML models has attracted increasing attention from clinicians to unveil the black box of ML models [23, 24]. This study explored subpopulations with heterogeneous treatment effects (HTE) of csCMVi and NRM after HSCT to establish survival trees that clinicians could easily interpret.

Furthermore, although time‐dependent covariates such as GVHD and competing risks such as relapse and NRM are frequently considered in statistical analyses in HSCT settings [25], the methodology to deal with both in ML has not been well elucidated. This study combined the weighting procedure [26] and the left‐truncated and right‐censored tree based on the classification and regression tree (LTRCART) algorithm [27] to fit survival data with time‐varying covariates and competing risks. This model successfully established survival trees that clinicians could easily interpret to predict the cumulative incidence of CMV reactivation and NRM after HSCT. We also evaluated the association between GVHD and the robustness of our analyses. In addition, we compared the accuracy of the ML algorithm with that of the Fine‐Gray model.

## 2 Methods

### 2.1 Patients

Clinical data were collected from the Transplant Registry Unified Management Program, a nationwide registry of the Japan Society for Transplantation and Cellular Therapy (JSTCT), and the Japanese Data Center for Hematopoietic Cell Transplantation. Patients diagnosed with acute myeloid leukemia, acute lymphoblastic leukemia, myelodysplastic syndromes, or chronic myeloid leukemia and who underwent the first HSCT between 2004 and 2016 between the ages of 18 and 70 were included in the study. Exclusion criteria and detailed transplant procedures have been described previously [1]. Patients with missing survival, relapse, or CMV treatment data were excluded from the analysis. Patients who received posttransplant cyclophosphamide for GVHD prophylaxis, those who could not achieve neutrophil engraftment or hematological complete remission after HSCT, and those who were administered anti‐CMV therapy before engraftment were also excluded. Generally, pp65 antigenemia surveillance was initiated during neutrophil engraftment. Polymerase chain reaction (PCR) testing for CMV was uncommon because of the public insurance system in Japan during the study period. csCMVi was defined as the initiation of CMV preemptive therapy as previously described [28]. Cord blood (CB) donors were considered negative for CMV. Performance status was evaluated according to the Eastern Cooperative Oncology Group criteria. Conditioning intensity was classified as previously described [29]. HLA were considered matched when the donor was serologically 8/8 matched for HLA‐A, ‐B, ‐C, and ‐DR. Disease risk was defined as previously reported [30]. In vivo T‐cell depletion was performed by administering anti‐thymocyte globulin, anti‐lymphocyte globulin, or alemtuzumab. When only patients who underwent bone marrow transplantation (BMT) or peripheral blood stem cell transplantation (PBSCT) were analyzed, CB was excluded from a covariate. NRM was defined as death without disease relapse or progression, considering relapse as a competing risk. This study was performed in accordance with the Declaration of Helsinki. It was approved by the Transplant Registry Unified Management Program's data management committee and the Tokyo Metropolitan Komagome Hospital ethics committee (approval number 1966) and written informed consent was obtained from all participants in this study.

### 2.2 Development of Machine Learning Algorithms

The cohort was divided into two groups: a training cohort (75%) and a test cohort (25%). The training cohort was used to develop the ML model. Functions crprep (mstate package) and LTRCART (LTRCtrees package) were sequentially applied to construct survival trees that incorporated competing risks and time‐varying covariates. The crprep function handles competing risks by assigning the “weights” in Geskus (2011) [26]. Following the idea of Geskus (2011), the LTRCART function [27] was applied to the weighted dataset since the LTRCART function allows for the inclusion of time‐dependent weights along with the left truncation and right censoring.

Survival trees for the cumulative incidence of csCMVi were created, treating GVHD as a time‐varying covariate and relapse and NRM as competing risks. When predicting the cumulative incidence of NRM, GVHD and csCMVi were treated as time‐dependent covariates, and relapse was treated as a competing risk. The complexity parameters were determined to avoid including too many leaves. In each analysis, survival trees were created 100 times with different random seeds to check their robustness against random variations, and the most frequently observed tree in each analysis was displayed in the main figure. Class imbalance was not adjusted in the ML model training. Our codes are available at GitHub so that readers can apply our framework in their settings.

A test cohort was used to evaluate the survival tree performance. The c‐indices of the survival tree and the Fine‐Gray model were compared. When applying the Fine‐Gray model, the same outcomes, covariates, time‐dependent covariates, and competing risks as those of the survival trees were adopted. Fine and Gray's method was used, and the impact of CMV reactivation on NRM was also evaluated considering the interaction terms of the candidate factors and CMV reactivation incidence in NRM prediction using csCMVi as a time‐dependent covariate, as previously reported [1]. The 95% CI for each C‐index was calculated using 200 bootstrap replicates.

When comparing the patient characteristics between training and test cohort, differences in numerical variables were compared with Wilcoxon rank sum test and those in categorical variables were compared using Fisher's exact test. R ver. 4.1.1 was used in this study.

## 3 Results

### 3.1 Patient Characteristics

Patient characteristics are shown in Table 1 and have been described in detail previously [1]. From 16,400 eligible patients, 10,480 patients with complete data were included. The median ages of recipients and donors at HSCT were 49 (range, 18–70) and 34 (range, 0–69; the age of the CB donor was considered 0), respectively. The cumulative incidence of csCMVi was 53.8% (95% confidence interval [CI]: 52.9–54.8) at 100 days after HSCT, and the median onset was 42 days after transplantation (interquartile range: 32–52 days). The overall survival, disease‐free survival, cumulative incidence of relapse, and NRM were 55.5% (95% CI: 54.4%–56.5%), 50.0% (95% CI: 49.0%–51.0%), 26.9% (95% CI: 26.0%–27.8%), and 23.1% (95% CI: 22.3%–24.0%), respectively, at 3 years after transplantation. Patient characteristics were generally similar between training cohort and test cohort (Table 1).

**TABLE 1:** Patient characteristics.

Characters	Patients (*n* = 10,480)	Training (*n* = 7860)	Patients (*n* = 2620)	*p* value
Primary disease				0.36
Acute myeloid leukemia	5811 (55.4%)	4344 (55.3%)	1467 (56.0%)	
Acute lymphoblastic leukemia	2466 (23.5%)	1873 (23.8%)	593 (22.6%)	
Myelodysplastic syndromes	1801 (17.2%)	1333 (17.0%)	468 (17.9%)	
Chronic myeloid leukemia	402 (3.8%)	310 (3.9%)	92 (3.5%)	
Patient age, median (range), years old	49 (18–70)	49 (18–70)	49 (18–70)	0.44
Donor median age (range), years old	33 (0–69)	33 (0–69)	33 (0–68)	0.33
Patient sex (male)				0.70
Male	6112 (58.3%)	4575 (58.2%)	1537 (58.7%)	
Female	4368 (41.7%)	3285 (41.8%)	1083 (41.3%)	
Donor sex (male)				0.66
Male	6318 (60.3%)	4748 (60.4%)	1570 (59.9%)	
Female	4162 (39.7%)	3112 (39.6%)	1050 (40.1%)	
Female donor to male recipient	2325 (22.2%)	1748 (22.2%)	583 (22.3%)	
Recipient CMV serology				0.30
Positive	8530 (81.4%)	6379 (81.2%)	2151 (82.1%)	
Negative	1950 (18.6%)	1481 (18.8%)	469 (17.9%)	
Donor CMV serology				0.96
Positive	5083 (48.5%)	3811 (48.5%)	1272 (48.5%)	
Negative	5397 (51.5%)	4049 (51.5%)	1348 (51.5%)	
Disease risk				0.036
High risk	4240 (40.5%)	3134 (39.9%)	1106 (42.2%)	
Standard risk	6240 (59.5%)	4726 (60.1%)	1514 (57.8%)	
Performance status (poor)				0.029
Good	9762 (93.1%)	7297 (92.8%)	2465 (94.1%)	
Poor	718 (6.9%)	563 (7.2%)	155 (5.9%)	
Stem cell source				0.55
Bone marrow	5920 (56.5%)	4418 (56.2%)	1502 (57.3%)	
Peripheral blood stem cell	1853 (17.7%)	1405 (17.9%)	448 (17.1%)	
Cord blood	2707 (25.8%)	2037 (25.9%)	670 (25.6%)	
Relation of the donor				0.81
Related donor	2582 (24.6%)	1932 (24.6%)	650 (24.8%)	
Unrelated donor	7898 (75.4%)	5928 (75.4%)	1970 (75.2%)	
HLA disparity				0.76
Match	4166 (39.8%)	3118 (39.7%)	1048 (40.0%)	
Mismatch	6314 (60.2%)	4742 (60.3%)	1572 (60.0%)	
Conditioning intensity				0.20
Myeloablative	7706 (73.5%)	5754 (73.2%)	1952 (74.5%)	
Reduced intensity	2774 (26.5%)	2106 (26.8%)	668 (25.5%)	
Total body irradiation				0.019
Yes	7521 (71.8%)	5688 (72.4%)	1833 (70.0%)	
No	2959 (28.2%)	2172 (27.6%)	787 (30.0%)	
Tacrolimus based GVHD prophylaxis				0.32
Yes	7352 (70.2%)	5534 (70.4%)	1818 (69.4%)	
No	3128 (29.8%)	2326 (29.6%)	802 (30.6%)	
Mycophenolate mofetil use				0.54
Yes	1154 (11.0%)	857 (10.9%)	297 (11.3%)	
No	9326 (89.0%)	7003 (89.1%)	2323 (88.7%)	
T‐cell depletion in vivo				0.072
Yes	872 (8.3%)	676 (8.6%)	196 (7.5%)	
No	9608 (91.7%)	7184 (91.4%)	2424 (92.5%)	
Transplant year				0.67
2004–2010	3878 (37.0%)	2918 (37.1%)	960 (36.6%)	
2011–2016	6602 (63.0%)	4942 (62.9%)	1660 (63.4%)	

*Note*: Data for patients with complete data were included.

Abbreviations: CMV, cytomegalovirus; GVHD, graft‐versus‐host disease; HLA, human leukocyte antigen.

### 3.2 Prediction of csCMVi

**FIGURE 1:**
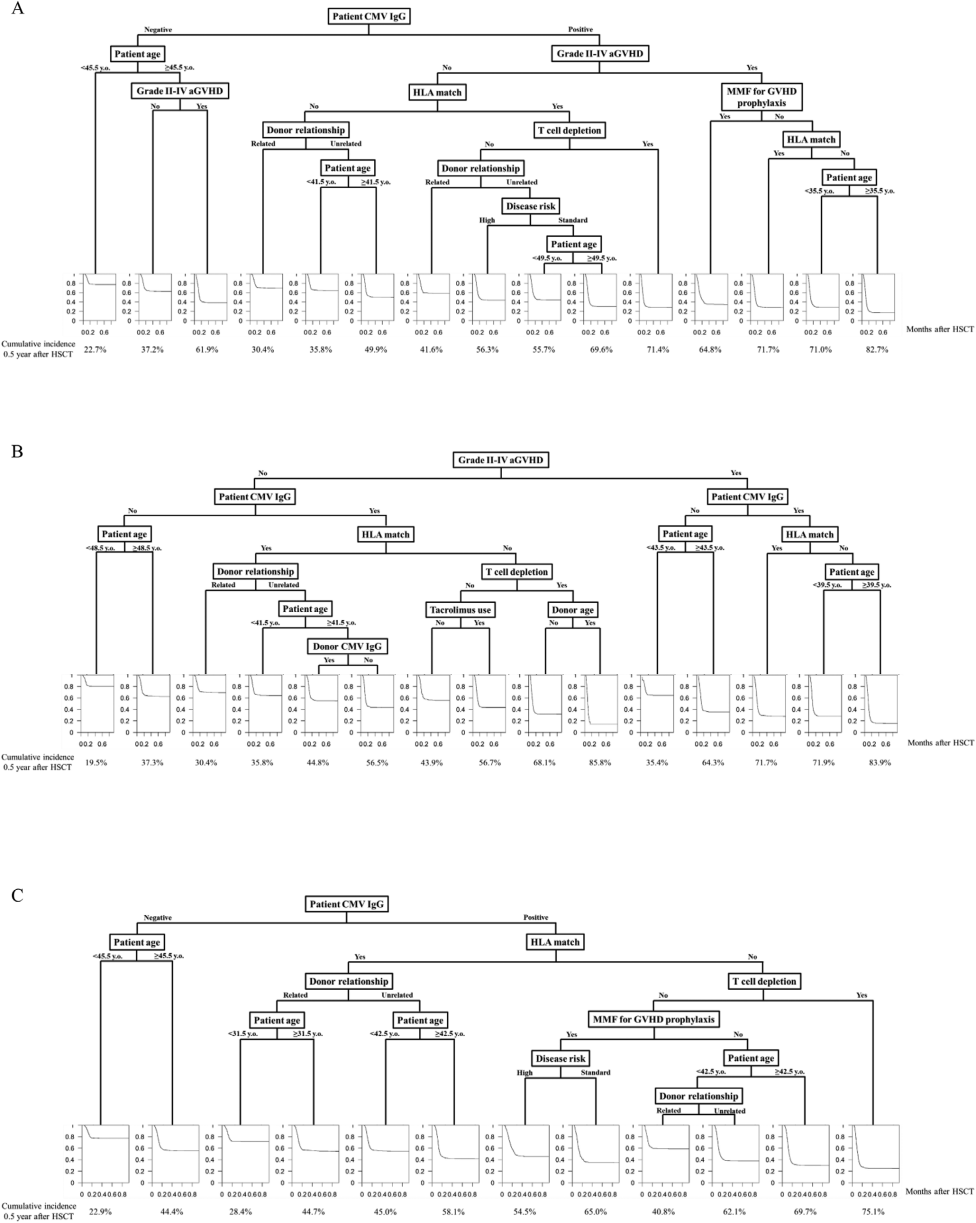
Survival trees to predict clinically significant cytomegalovirus infection (csCMVi) after allogeneic hematopoietic stem cell transplantation. Prediction of csCMVi (A) with graft‐versus‐host disease (GVHD) as a time‐dependent covariate, (B) only in patients who underwent bone marrow or peripheral blood stem cell transplant, and (C) only with pretransplant risk factors, excluding GVHD. The terminal node of the survival tree displays the fitted cumulative incidence curve for csCMVi in that node. Horizon axis, years from transplant. Cumulative incidence of csCMVi at 0.5 year after transplantation was shown below each curve.

First, we predicted CMV reactivation using GVHD as a time‐dependent covariate. The same tree was seen 86 out of 100 times (Figure 1A); in contrast, other survival trees were broadly similar (Figure S1). As shown in Figure 1A, patients were classified according to the CMV reactivation risk, and patient CMV IgG positivity was the most important predictor of csCMVi. In addition, the patients were successfully classified according to the estimated cumulative incidence of csCMVi, which was 22.7% at 0.5 year after HSCT in the lowest group and 82.7% in the highest group (Figure 1A).

Next, csCMVi was predicted in patients who underwent BMT or PBSCT because CB recipients have distinct characteristics for csCMVi [1]. An identical survival tree was observed 44 out of 100 times (Figure 1B and Figure S2). Although the most important predictor was the existence of Grades II–IV acute GVHD and not patient CMV IgG, the essential predictors were mostly the same as those shown in Figure 1A. The patients were classified based on the estimated cumulative incidence of csCMVi, which was 19.5% at 0.5 year after HSCT in the lowest group and 83.9% in the highest group (Figure 1B).

As shown in Figure 1A, Grades II–IV acute GVHD was an important predictor of csCMVi. However, GVHD, a post‐HSCT event, is not useful for determining the indication for CMV prophylaxis because the administration of CMV prophylactic agents, such as letermovir, is generally initiated at the time of HSCT. Therefore, csCMVi was predicted, excluding GVHD. The same tree was depicted 91 out of 100 times (Figure 1C and Figure S3). Patient CMV IgG positivity, transplantation from a human leukocyte antigen (HLA)‐mismatched donor, and older patient age at HSCT were identified as important predictors of csCMVi. The cases were classified according to the estimated cumulative incidence of csCMVi, which was 22.9% at 0.5 year after HSCT in the lowest group and 75.1% in the highest group.

### 3.3 Prediction of NRM

**FIGURE 2:**
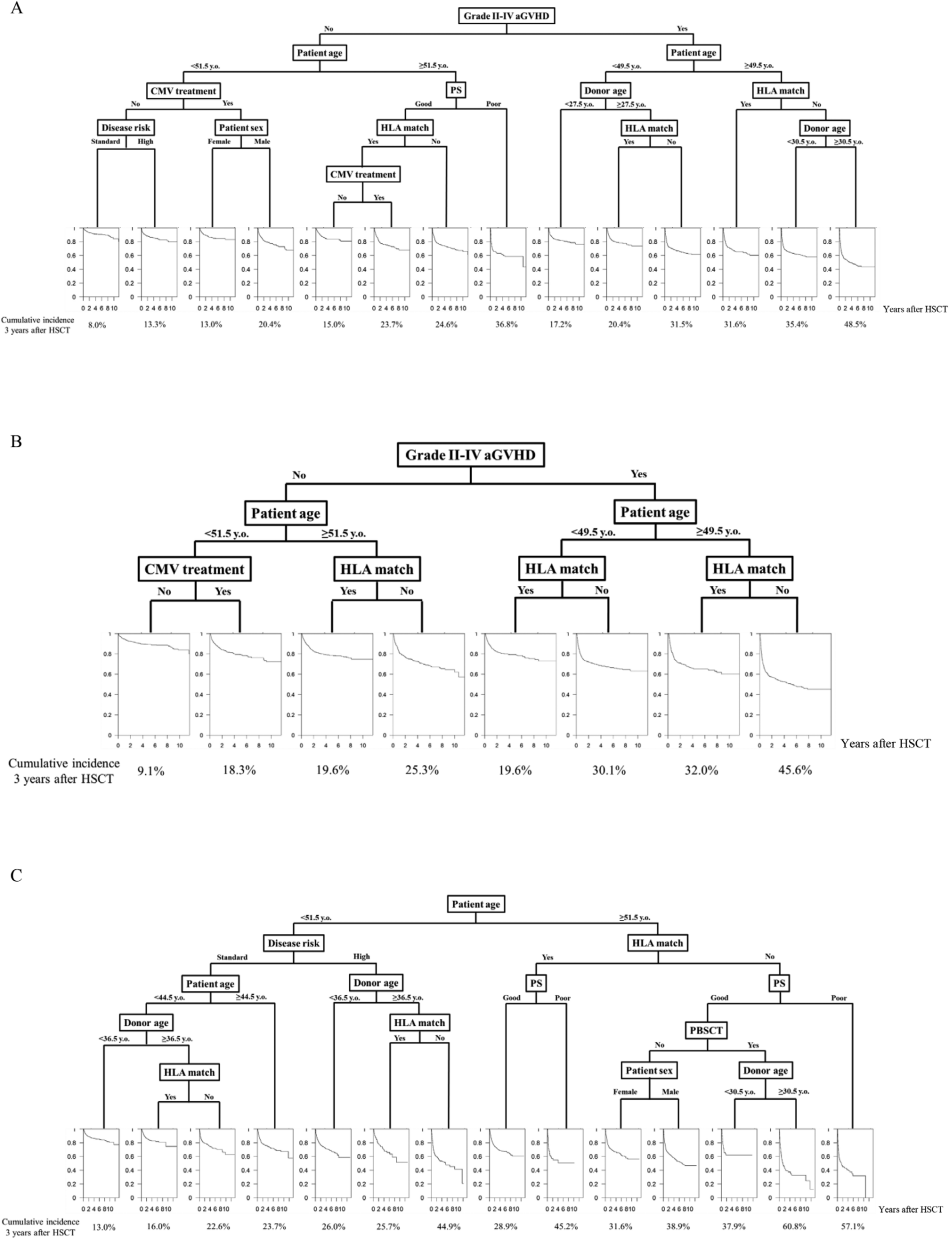
Survival trees to predict non‐relapse mortality (NRM) after allogeneic hematopoietic stem cell transplantation. Prediction of NRM (A) with clinically significant cytomegalovirus infection (csCMVi) and graft‐versus‐host disease (GVHD) as time‐dependent covariates, (B) only in patients who underwent bone marrow or peripheral blood stem cell transplant, and (C) only with pretransplant risk factors, excluding csCMVi and GVHD. The terminal node of the survival tree displays the fitted cumulative incidence curve for NRM in that node. Horizon axis, years from transplant. Cumulative incidence of NRM at 3 years after transplantation was shown below each curve.

We also predicted NRM using GVHD and csCMVi as time‐dependent covariates. Among 100 repetitions, the same survival tree was constructed 53 times (Figure 2A and Figure S4). The patients were successfully classified according to the estimated cumulative incidence of NRM, which was 8.0% at 3 years after HSCT in the lowest group and 48.5% in the highest group.

We also predicted NRM only in patients who underwent BMT or PBSCT, similar to the prediction of csCMVi. The same survival tree was observed 85 out of 100 times (Figure 2B and Figure S5). In this setting, the tree became simple based on the variation in complexity parameters (data not shown). The patients were classified based on the estimated cumulative incidence of NRM, which was 9.1% at 3 years after HSCT in the lowest group and 45.6% in the highest group.

We then predicted NRM using only pre‐HSCT factors, excluding csCMVi and Grades II–IV acute GVHD. Although the most common survival tree was depicted only 39 out of 100 times, the other trees were generally similar to the most popular tree (Figure 2C and Figure S6). Older patient age was the most important predictor, followed by HLA disparity and high‐disease risk. The cases were classified according to the estimated cumulative incidence of NRM, which was 13.0% at 3 years after HSCT in the lowest group and 57.1% in the highest group.

### 3.4 Comparison of ML and Fine‐Gray Model

Finally, we evaluated the performance of the ML models by comparing their c‐indices with those of the conventional Fine‐Gray model using the test cohort (Table 2 and Tables S1–S6). We repeated the validation 200 times in each setting, and the mean c‐index of csCMVi prediction without GVHD was 0.624 (95% CI: 0.613–0.639) in Fine‐Gray model and 0.618 (95% CI: 0.606–0.630) in the survival tree. In addition, the mean c‐index of NRM prediction without GVHD or csCMVi was 0.648 (0.629–0.667) in the Fine‐Gray model and 0.628 (0.608–0.644) in the survival tree. Likewise, the c‐indices between the conventional model and ML were not significantly different in csCMVi and NRM predictions in all settings, which suggested a comparable predictive performance between the two methods (Table 2). In addition, covariates which were picked up in the ML model and those identified in Fine‐Gray model were highly overlapped, suggesting that our ML model was reasonable. Among the statistically significant factors in Fine‐Gray model, 61.5%, 77.8%, 58.3%, 77.8%, 33.3%, and 50.0% were also included in the ML model in each situation. On the other hand, among the non‐significant factors in Fine‐Gray model, 100.0%, 84.6%, 100.0%, 93.3%, 92.9%, and 100.0% were not included in the ML model (Figures 1 and 2 and Tables S1–S6).

**TABLE 2:** Comparison of c‐index according to statistical methods.

Event	Model	with GVHD	w GVHD/wo CB	wo GVHD	wo GVHD/wo CMV
csCMVi	Fine‐Gray	0.647	0.661	0.624	NA
		(0.633–0.660)	(0.645–0.674)	(0.613–0.639)	NA
	CART	0.644	0.654	0.618	NA
		(0.630–0.656)	(0.638–0.670)	(0.606–0.630)	NA
NRM	Fine‐Gray	0.666	0.674	0.651	0.648
		(0.644–0.685)	(0.654–0.696)	(0.633–0.671)	(0.629–0.667)
	CART	0.641	0.645	0.625	0.628
		(0.619–0.661)	(0.623–0.669)	(0.603–0.643)	(0.608–0.644)

*Note*: Mean c‐indices and 95% confidence interval were shown.

Abbreviations: CART, classification and regression tree; CMV, cytomegalovirus; csCMVi, clinically significant cytomegalovirus infection; GVHD, graft‐versus‐host disease; NRM, non‐relapse mortality; w, with; wo, without.

## 4 Discussion

CMV infections, directly and indirectly, increase non‐relapse deaths among HSCT recipients, and prophylaxis with letermovir can effectively suppress csCMVi and probably decrease NRM in CMV‐seropositive transplant recipients [29, 31]. However, considering the side effects of letermovir, possible resistant infections, and high‐medical costs, more sophisticated patient selection for letermovir prophylaxis is craved. Although various recent attempts have been made to apply ML to hematology and HSCT, such algorithms still need to be sufficiently implemented in daily practice, partially owing to their limited transparency and interpretability [23]. Eisenberg et al. published an ML algorithm to predict early CMV reactivation and death after HSCT, which provided the SHAP score to assure explainability [32]—transparency and interpretability have become more important in ML, especially in clinical settings [33]. In addition, although time‐dependent covariates and competing risks are frequently handled in statistical analyses in HSCT settings [25], such as GVHD as a time‐dependent covariate, and relapse and NRM as competing risks, dealing with both concurrently has yet to be well elucidated in ML. In this study, our methods successfully fit survival data with time‐dependent covariates and competing risks, and the depicted survival trees were highly interpretable [34]. Our results could contribute to identification of subpopulations with treatment effects of csCMVi, who should preferentially receive letermovir prophylaxis. Although cost‐effectiveness of letermovir has been reported [35], narrowing down the target population for prophylaxis further improve the cost‐benefit balance.

Previous studies have identified various risk factors associated with CMV reactivation and NRM after HSCT [1, 2, 13, 36, 37, 38]. Important risk factors in our study were generally similar to these already‐known risk factors, which suggests the appropriateness of our model. Among them, T‐cell depletion, an important risk factor for csCMVi [1, 7], seemed significant solely at the branch of HLA‐mismatched transplant in our study (Figure 1A–C). Such hierarchical effect has not been reported in the previous studies about CMV infection with or without ML [11, 32, 39], and further investigation is necessary considering the reduction of GVHD severity and NRM via T‐cell depletion in GVHD prophylaxis. In addition, considering the importance of CMV viral load kinetics [39, 40], anti‐CMV immune reconstitution [41] and chronological transition of other biomarkers [42, 43, 44], handling more complicated information such as longitudinal data [45, 46] may allow more precise HTE group identification and might even enable discriminating spontaneous clearance [47], abortive infection [48], breakthrough infection [49], and CMV disease [39].

Although relapse risk was not a goal of our study, relapse of the primary hematological disease is also an important barrier after HSCT [50]. The association between CMV reactivation and relapse risk is controversial [11] and may be dependent on clinical situation such as type of disease, transplantation setting, and use of anti‐thymocyte globulin [2, 6, 28]. Our methodology might be useful to identify subpopulations with HTEs of csCMVi on relapse, and perhaps ML can be also applied to characterize T‐cell receptor repertoire after CMV reactivation which might be important for preventing repetitive infection and/or graft‐versus‐leukemia effect [51, 52].

Analyses of the c‐indices further validated the performance of the survival trees. Most picked‐up factors in the ML model were also significant in Fine‐Gray model, suggesting the reliability of our ML model. The low “sensitivity” and high “specificity” of the covariates selected in the ML model, considering the Fine‐Gray model as a benchmark, implies more conservative selection of the covariates in the ML model than that in the Fine‐Gray model. The ML model should have controlled the false subgroup discovery rate better than the Fine‐Gray model because the statistical significance in the Fine‐Gray model was not adjusted for multiple comparisons. Thus, the HTE groups suggested by the ML model are more likely to be reproduced in independent data than the Fine‐Gray model. In csCMVi prediction, the c‐index in the without‐GVHD setting was lower than that when GVHD was included as a time‐dependent covariate, which suggested the importance of GVHD as a risk factor for csCMVi, as in previous reports [2, 11]. Although integrating post‐HSCT factors is a promising strategy to improve predictive performance, as reported by Eisenberg et al. [32], the indication for letermovir is generally determined at the time of HSCT and therefore prediction solely with pre‐HSCT factors is also clinically important. Adopting LTRC forest model is another way, which can substantially improve prediction accuracy compared with tree‐based model [53], although interpretability might degrade. These challenges might be addressed in the future by the development of novel ML models with enhanced accuracy. In addition, higher c‐indexes were seen when CB recipients were excluded. This difference in the c‐index with or without CBT was similar to the NRM prediction. This might be due to the different transplantation settings and frequencies of various complications in CBT compared to BMT and PBSCT [54, 55, 56], and different prediction strategies against csCMVi and NRM may be necessary for CBT.

Our study had some limitations. First, CMV antigenemia testing was performed to detect CMV reactivation. Although the comparative preventive efficacy of CMV disease between antigenemia and PCR monitoring has been reported [57], PCR assays can be more sensitive than CMV antigenemia testing [11], and the timing of starting preemptive therapy might be affected by the detection technique, which may have influenced the results. Second, this retrospective study did not include patients receiving CMV prophylaxis because we believe patients with HTEs of csCMVi are the true candidates for CMV prophylaxis. However, considering csCMVi during letermovir prophylaxis [49] and after letermovir cessation [58], along with recent reports about the efficacy and safety of extended‐duration letermovir prophylaxis [59], accumulation of data with letermovir prophylaxis and prospective validation is also warranted. Third, the long inclusion period also might affect the results, although CMV preemptive strategy was mostly same in the inclusion period.

In conclusion, our study suggested subpopulations with HTEs of csCMVi and NRM after HSCT by concomitantly fitting survival data with time‐varying covariates and competing risks and established interpretable survival trees with valid performance. We believe that this methodology will be useful for predicting various events after HSCT.

## Author Contributions

T.T., Y.N., K.H., S.K., T.N., M.O., and A.T. designed the study. T.T. and K.H. wrote the manuscript. Y.N. analyzed the data. K.H. supervised data management. N.U., N.S., W.T., T.N., Y.O., M.T., S.Y., Y.K., T.E., M.S., S.O., H.O., S.T., K.K., J.K., T.F., and Y.A. contributed to data collection. M.O. and Y.A. supervised the research. All authors reviewed and approved the manuscript.

## Conflicts of Interest

The authors declare no conflicts of interest.

## Supporting information




**Supplemental Figure 1**: Survival tree for predicting clinically significant cytomegalovirus infection (csCMVi) after allogeneic hematopoietic stem cell transplantation with graft‐versus‐host disease as a time‐dependent covariate. An identical survival tree was observed 13 times out of 100. Cumulative incidence of csCMVi at 0.5 year after transplantation was shown below each curve. **Supplemental Figure 2**: Survival trees were used to predict clinically significant cytomegalovirus infection (csCMVi) after allogeneic hematopoietic stem cell transplantation with graft‐versus‐host disease as a time‐dependent covariate in patients who underwent bone marrow or peripheral blood stem cell transplantation. (A, B) Identical survival trees were observed 23 and 16 times out of 100 each.Cumulative incidence of csCMVi at 0.5 year after transplantation was shown below each curve. **Supplemental Figure 3**: A survival tree to predict clinically significant cytomegalovirus infection (csCMVi) after allogeneic hematopoietic stem cell transplantation using only pre‐transplant factors (without graft‐versus‐host disease). An identical survival tree was observed eight times out of 100.Cumulative incidence of csCMVi at 0.5 year after transplantation was shown below each curve. **Supplemental Figure 4**: A survival tree to predict non‐relapse mortality (NRM) after allogeneic hematopoietic stem cell transplantation with graft‐versus‐host disease and clinically significant cytomegalovirus infction as time‐dependent covariates. An identical survival tree was observed 46 times out of 100. Cumulative incidence of NRM at 3 years after transplantation was shown below each curve. **Supplemental Figure 5**: A survival tree for predicting non‐relapse mortality (NRM) after bone marrow or peripheral blood stem cell transplantation, with graft‐versus‐host disease and clinically significant cytomegalovirus infection as time‐dependent covariates. An identical survival tree was observed 11 times out of 100. Cumulative incidence of NRM at 3 years after transplantation was shown below each curve. **Supplemental Figure 6**: Survival trees to predict non‐relapse mortality (NRM) after allogeneic hematopoietic stem cell transplantation with only pre‐transplant factors (without graft‐versus‐host disease or clinically significant cytomegalovirus infection). (A‐C) Identical survival trees were observed 27, nine, and nine times out of 100 each. Cumulative incidence of NRM at 3 years after transplantation was shown below each curve.

**Supporting File 2**: jha270117‐sup‐0002‐tableS1‐S6.xlsx

## Data Availability

The data that support the findings of this study are available from the corresponding author (Takashi Toya) upon reasonable request. In addition, the ML code we used is available at GitHub (https://github.com/KomaNakajima/GVHD_analysis.git).
