# Supplementary material for: Machine Learning Algorithm to Explore Patients With Heterogeneous Treatment Effects of Clinically Significant CMV Infection and Non‐Relapse Mortality After HSCT
Source: EJHaem. 2025 Aug 9;6(4):e70117. doi: 10.1002/jha2.70117 (PMC12335206; doi:10.1002/jha2.70117)
Supplement: Supplementary file 1 — Supplemental Figure 1: Survival tree for predicting clinically significant cytomegalovirus infection (csCMVi) after allogeneic hematopoietic stem cell transplantation with graft‐versus‐host disease as a time‐dependent covariate. An identical survival tree was observed 13 times out of 100. Cumulative incidence of csCMVi at 0.5 year after transplantation was shown below each curve. Supplemental Figure 2: Survival trees were used to predict clinically significant cytomegalovirus infection (csCMVi) after allogeneic hematopoietic stem cell transplantation with graft‐versus‐host disease as a time‐dependent covariate in patients who underwent bone marrow or peripheral blood stem cell transplantation. (A, B) Identical survival trees were observed 23 and 16 times out of 100 each.Cumulative incidence of csCMVi at 0.5 year after transplantation was shown below each curve. Supplemental Figure 3: A survival tree to predict clinically significant cytomegalovirus infection (csCMVi) after allogeneic hematopoietic stem cell transplantation using only pre‐transplant factors (without graft‐versus‐host disease). An identical survival tree was observed eight times out of 100.Cumulative incidence of csCMVi at 0.5 year after transplantation was shown below each curve. Supplemental Figure 4: A survival tree to predict non‐relapse mortality (NRM) after allogeneic hematopoietic stem cell transplantation with graft‐versus‐host disease and clinically significant cytomegalovirus infction as time‐dependent covariates. An identical survival tree was observed 46 times out of 100. Cumulative incidence of NRM at 3 years after transplantation was shown below each curve. Supplemental Figure 5: A survival tree for predicting non‐relapse mortality (NRM) after bone marrow or peripheral blood stem cell transplantation, with graft‐versus‐host disease and clinically significant cytomegalovirus infection as time‐dependent covariates. An identical survival tree was observed 11 times out of 100. Cumulative inc [file JHA2-6-e70117-s001.pdf]

Supplemental Figure 1

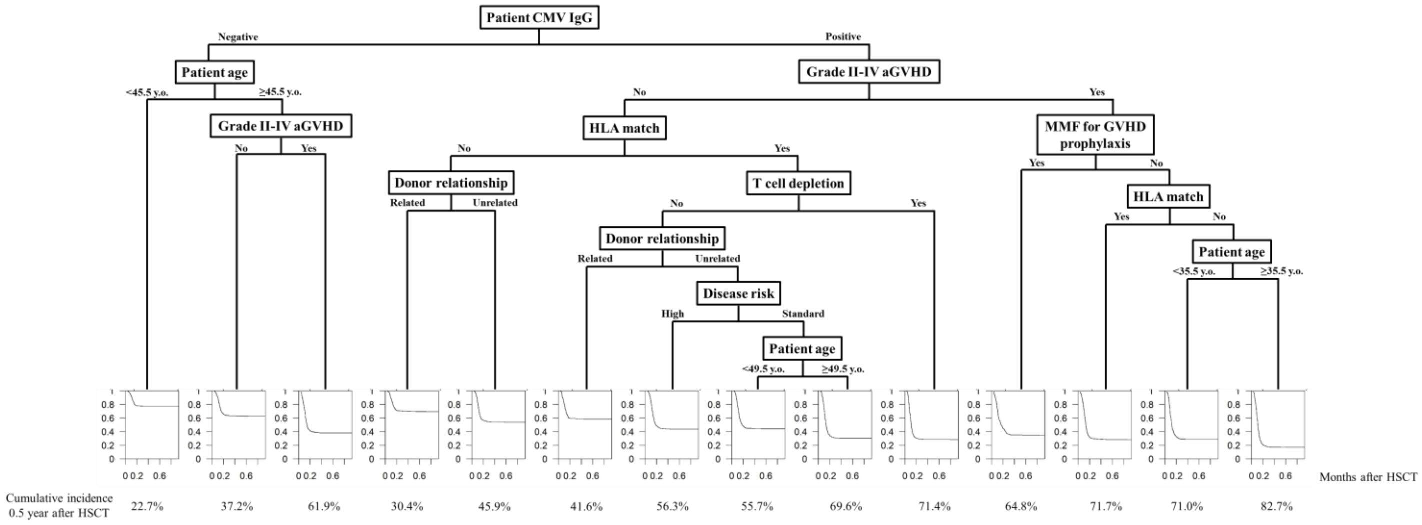

Supplemental Figure 1. Survival tree for predicting clinically significant cytomegalovirus infection (csCMVi) after allogeneic hematopoietic stem cell transplantation with graft-versus-host disease as a time-dependent covariate. An identical survival tree was observed 13 times out of 100. Cumulative incidence of csCMVi at 0.5 year after transplantation was shown below each curve.

Supplemental Figure 2

A

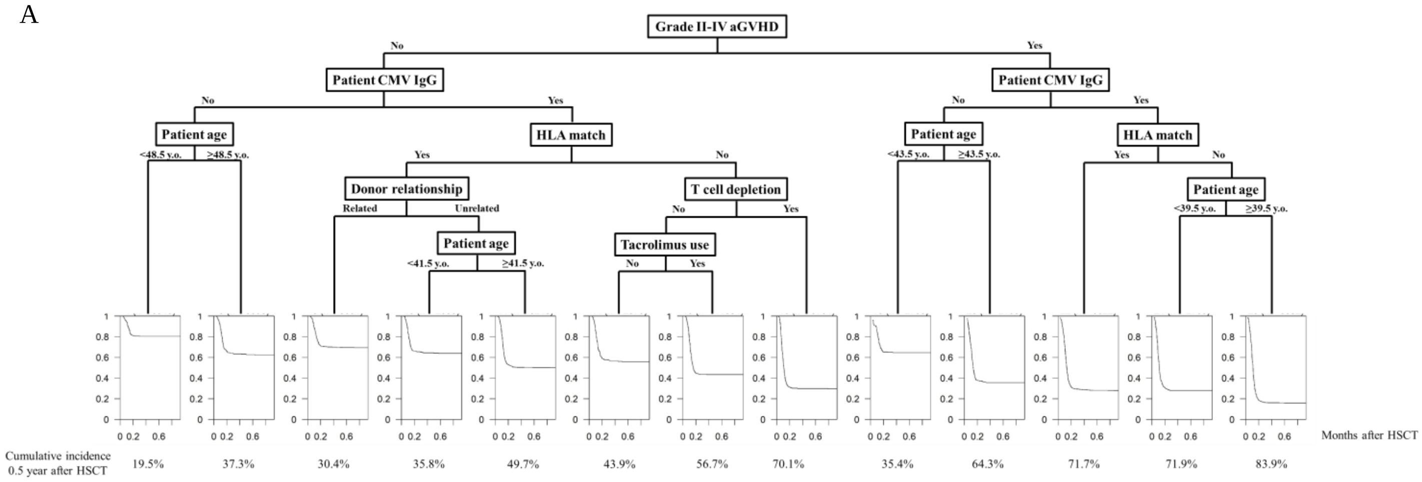

B

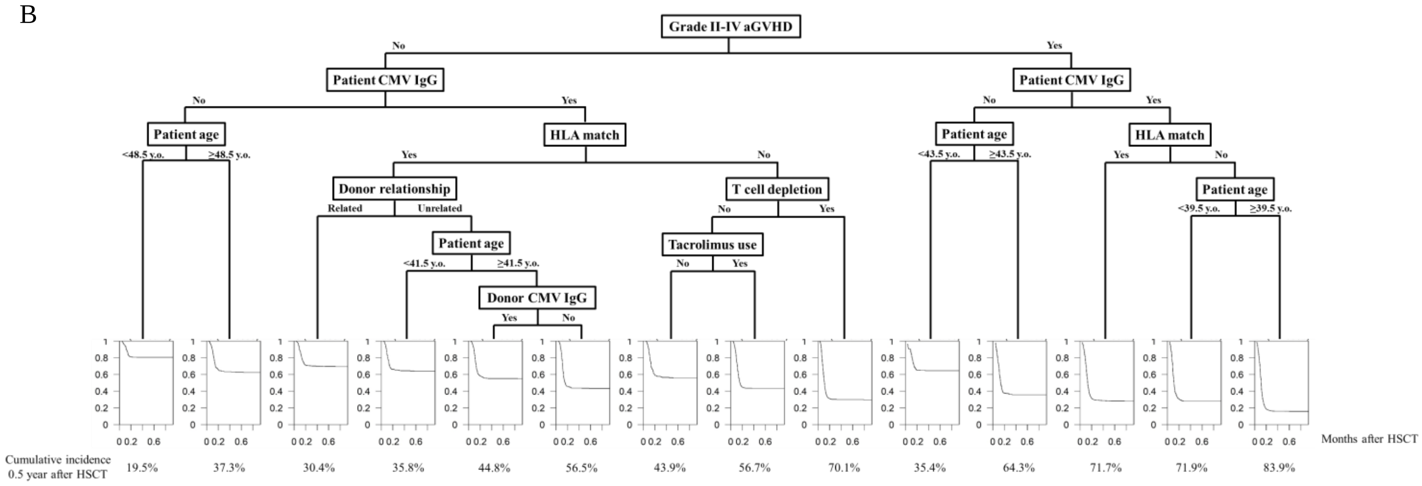

Supplemental Figure 2. Survival trees were used to predict clinically significant cytomegalovirus infection (csCMVi) after allogeneic hematopoietic stem cell transplantation with graft-versus-host disease as a time-dependent covariate in patients who underwent bone marrow or peripheral blood stem cell transplantation. (A, B) Identical survival trees were observed 23 and 16 times out of 100 each. Cumulative incidence of csCMVi at 0.5 year after transplantation was shown below each curve.

Supplemental Figure 3

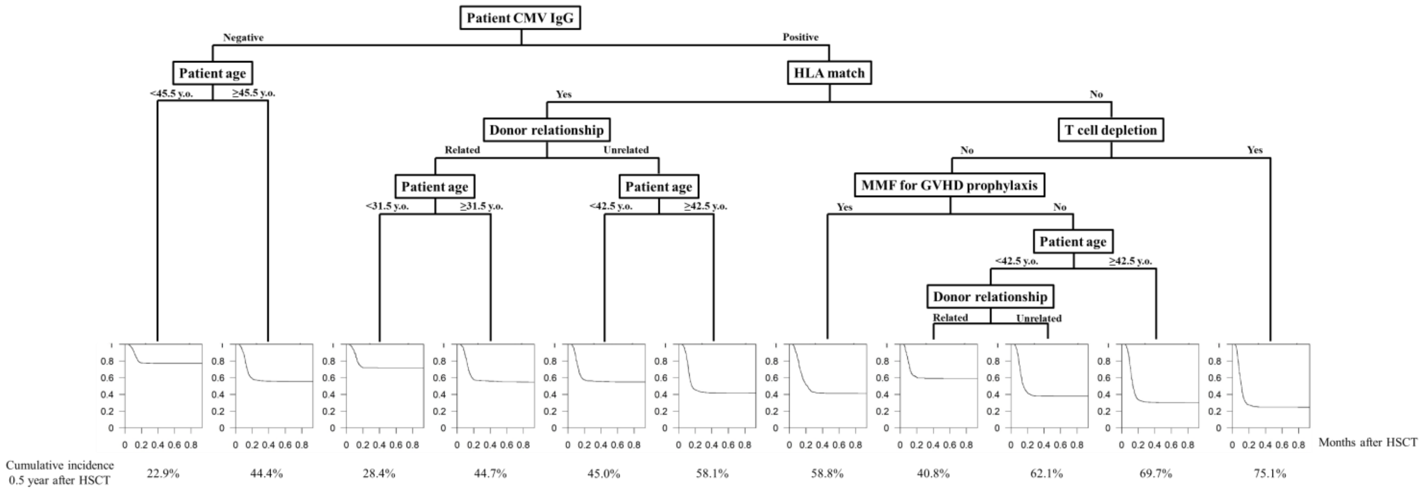

Supplemental Figure 3. A survival tree to predict clinically significant cytomegalovirus infection (csCMVi) after allogeneic hematopoietic stem cell transplantation using only pre-transplant factors (without graft-versus-host disease). An identical survival tree was observed eight times out of 100. Cumulative incidence of csCMVi at 0.5 year after transplantation was shown below each curve.

Supplemental Figure 4

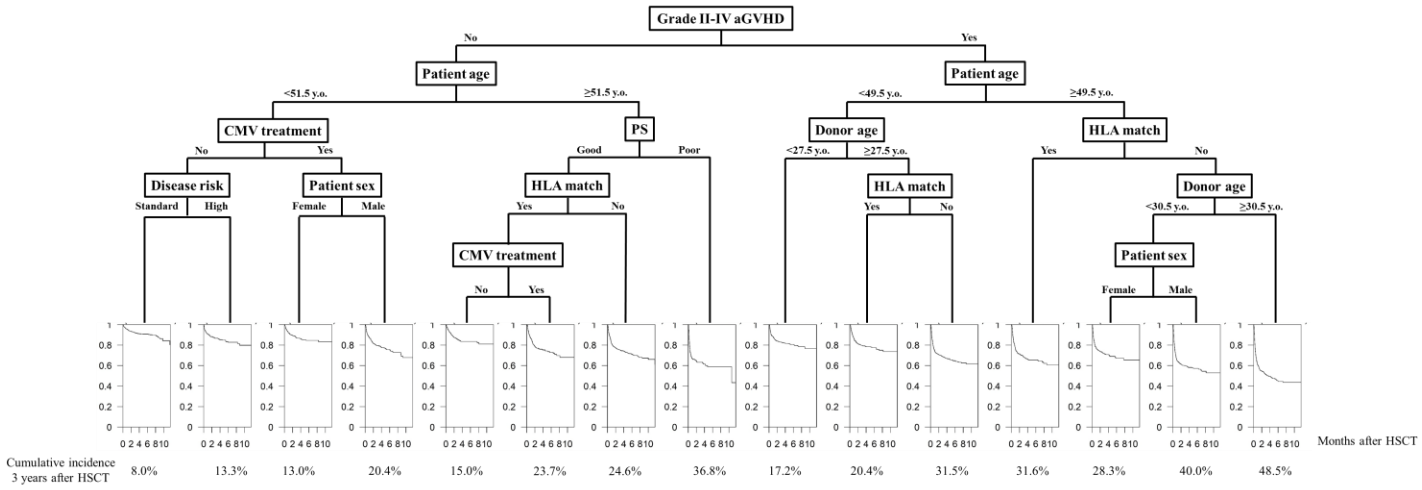

Supplemental Figure 4. A survival tree to predict non-relapse mortality (NRM) after allogeneic hematopoietic stem cell transplantation with graft-versus-host disease and clinically significant cytomegalovirus infection as time-dependent covariates. An identical survival tree was observed 46 times out of 100. Cumulative incidence of NRM at 3 years after transplantation was shown below each curve.

Supplemental Figure 5

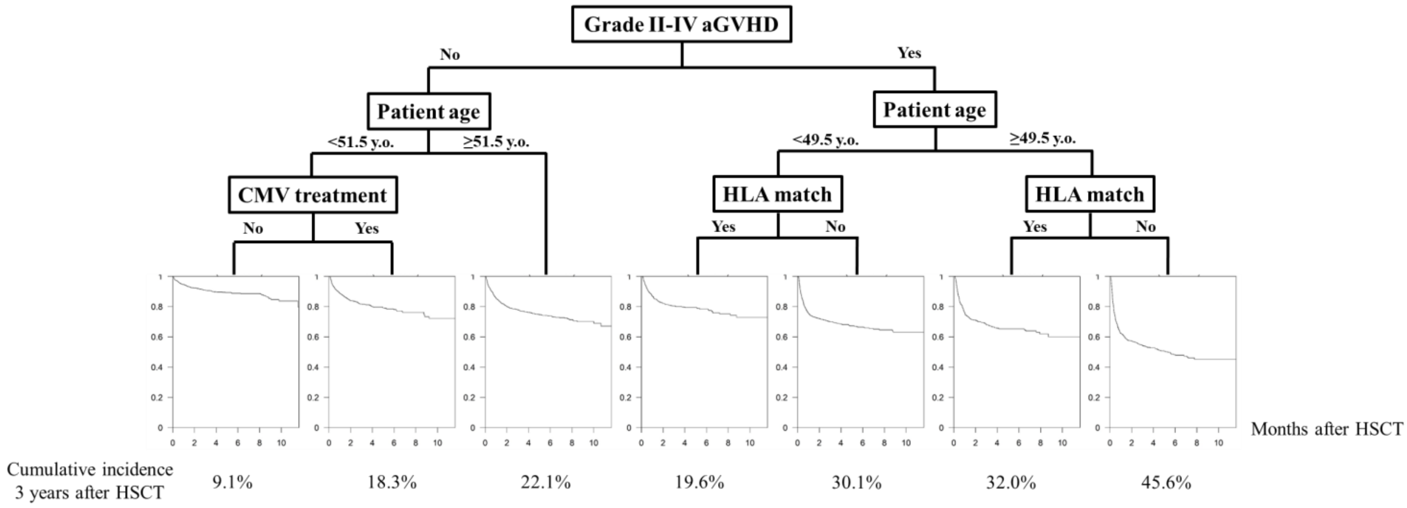

Supplemental Figure 5. A survival tree for predicting non-relapse mortality (NRM) after bone marrow or peripheral blood stem cell transplantation, with graft-versus-host disease and clinically significant cytomegalovirus infection as time-dependent covariates. An identical survival tree was observed 11 times out of 100. Cumulative incidence of NRM at 3 years after transplantation was shown below each curve.

Supplemental Figure 6

A

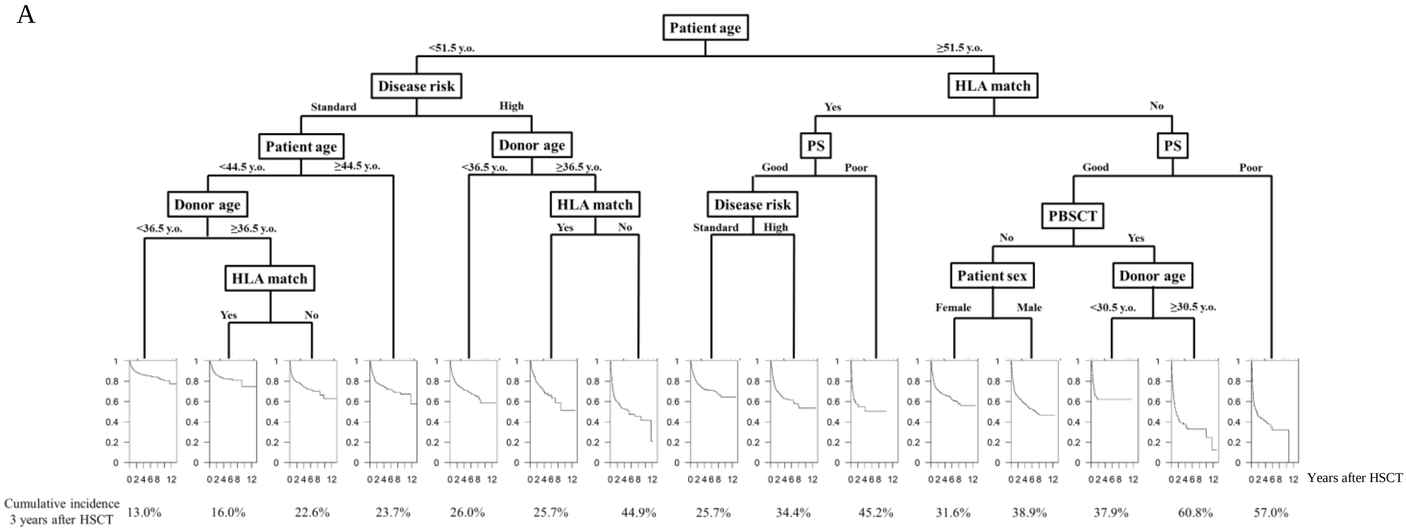

B

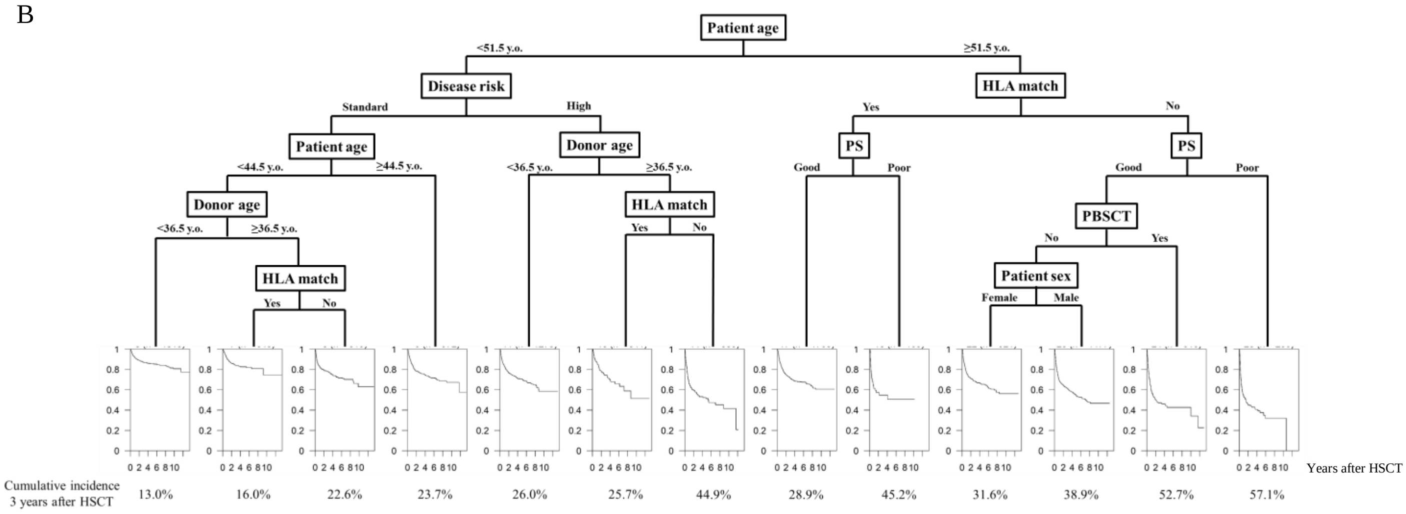

C

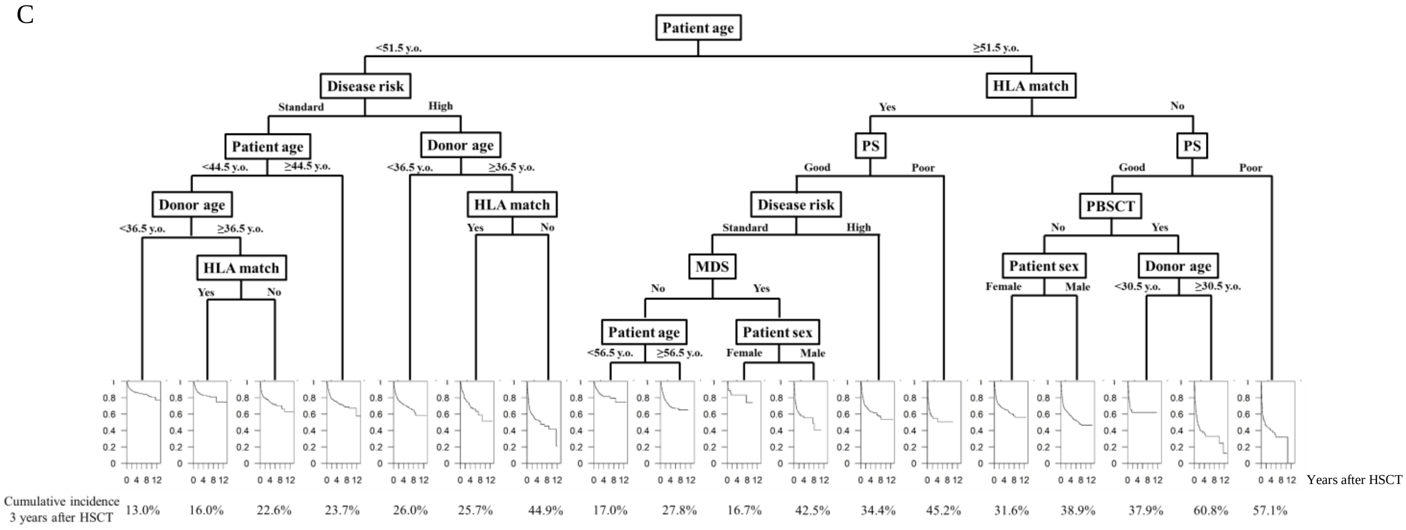

Supplemental Figure 6. Survival trees to predict non-relapse mortality (NRM) after allogeneic hematopoietic stem cell transplantation with only pre-transplant factors (without graft-versus-host disease or clinically significant cytomegalovirus infection). (A-C) Identical survival trees were observed 27, nine, and nine times out of 100 each. Cumulative incidence of NRM at 3 years after transplantation was shown below each curve.
